# Toxicological Profile of Plasmonic Nanoparticles in Zebrafish Model

**DOI:** 10.3390/ijms22126372

**Published:** 2021-06-14

**Authors:** Marta d’Amora, Vittoria Raffa, Francesco De Angelis, Francesco Tantussi

**Affiliations:** 1 Istituto Italiano di Tecnologia, Via Morego 30, 16163 Genova, Italy; francesco.deangelis@iit.it (F.D.A.); francesco.tantussi@iit.it (F.T.); 2 Department of Biology, University of Pisa, S.S. 12 Abetone e Brennero 4, 56127 Pisa, Italy; vittoria.raffa@unipi.it

**Keywords:** plasmonic nanoparticles, toxicity, zebrafish, development, adulthood, gold, silver

## Abstract

Plasmonic nanoparticles are increasingly employed in several fields, thanks to their unique, promising properties. In particular, these particles exhibit a surface plasmon resonance combined with outstanding absorption and scattering properties. They are also easy to synthesize and functionalize, making them ideal for nanotechnology applications. However, the physicochemical properties of these nanoparticles can make them potentially toxic, even if their bulk metallic forms are almost inert. In this review, we aim to provide a more comprehensive understanding of the potential adverse effects of plasmonic nanoparticles in zebrafish (*Danio rerio*) during both development and adulthood, focusing our attention on the most common materials used, i.e., gold and silver.

## 1. Introduction

Plasmonic nanoparticles possess unique catalytic, electronic, and optical properties (absorption, scattering, and field enhancement) related to localized surface plasmon resonance (LSPR) [1,2]. Moreover, they have a highly stable nature and high surface area, and they can be easily and rapidly synthesized and functionalized with different biomolecules. Gold and silver are the most widely and commonly used materials for plasmonic nanoparticle fabrication because they can finely tune their plasmonic response, covering a broad-spectrum range from the ultraviolet to the near-infrared regions. The position and width of their resonance depend on several parameters, such as shape, dispersion, size, surface coating, and uniformity [3,4,5,6]. Thanks to their features, gold (Au-NPs) and silver nanoparticles (Ag-NPs) are employed in a wide range of fields, including environmental science, catalysis, the food industry (food packaging, storage, additives), bioimaging and biosensing, [7,8] and nanomedicine [9,10].

The constant increase in the use of gold and silver nanoparticles and their consequent release in the environment can lead to potential risks for human health and ecosystems. A route for their successful and safe application consists in the careful investigation of their toxicological profiles. Such studies have been conducted so far using different models, revealing the possible toxic effects of gold and silver nanoparticles, both in vitro and in vivo. Most nanotoxicological works have been carried out by using different cell lines to investigate the uptake and the intracellular localization of gold and silver nanoparticles and their effects in terms of DNA damage, apoptosis, genotoxicity, and alteration of the immune system. However, the findings obtained from these studies have limited translational value due to the poor correlation between in vitro and in vivo mechanisms and systems [11,12]. Plated cells cannot reproduce the high complexity that takes place in the whole organism concerning the assimilation, localization, biotransformation, and clearance. Consequently, in vivo assessments are essential to accurately investigate the fate and effects of gold and silver nanoparticles. Even if both studies are important to fully understand the toxicological profile of Au-NPs and Ag-NPs, the in vivo screenings are more predictive of risks for humans.

Recently, zebrafish (*Danio rerio*) have been employed as alternative and powerful in vivo models to assess the biosafety of different nanoparticles. These vertebrate organisms are the most appropriate models for this type of research thanks to their great relevance to human genetics [13,14] and several peculiar features and advantages. Zebrafish are small and possess a high fecundity and an external and quick development. They mature to adulthood in 3 months [15], while all the larvae organs are completely formed within 5 dpf (days post fertilization) [16]. The developing embryos are optically transparent, allowing for an instant visualization of the cells and the organogenesis [17,18]. Consequently, malformations induced by nanoparticles in several tissues and organs (i.e., notochord, brain, heart, yok sac) can be easily and directly visualized and examined by using a stereomicroscope. Moreover, the digestive, nervous, and cardiovascular systems of zebrafish are comparable to mammals [19]. Additionally, zebrafish can be easily maintained and handled. Zebrafish have the ability to show the mechanism of developmental toxicity thanks to their analogy with mammals [12].

This in-depth review comprehensively describes the present knowledge on the adverse effects and biointeractions of gold and silver nanoparticles in zebrafish during the development and in the adult phase. We aim to provide insight into the behavior of these nanoparticles in a vertebrate system, with consideration of the nanoparticle’s physicochemical properties and other factors that can influence their toxicity.

## 2. Gold Nanoparticles

Currently, gold nanoparticles are increasingly employed in different industrial and scientific areas, such as catalysis, electronics, and medicine, thanks to their compatibility, nonimmunogenicity, and easy and well-established processes of synthesis and functionalization with specific and selective molecules (e.g., peptides, fluorophores, and antibodies) [20]. Au-NPs are suitable as drug delivery systems [21,22,23,24] and diagnostic agents [25,26] for the diagnosis and treatment of different illnesses, including AIDS, diabetes [27], Alzheimer’s, [28] and rheumatoid arthritis [29]. Moreover, gold nanoparticles are applied in hyperthermia for cancer treatment, due to their capability to convert absorbed light into heat [4,30]. Gold particles can also be used as amplification media in surface-enhanced Raman spectroscopy (SERS) and as contrast elements in optical microscopy [31,32] and biosensing.

While gold in its bulk state is inert, gold nanoparticles possess higher reactivity and a great surface area to volume ratio [33,34,35]. Such features allow the interplay with biological environments possibly leading to toxicity. In the past years, several toxicological screenings of Au-NPs have been carried out in vitro and in vivo on different cell types and living organisms to ensure efficient and safe application of these nanomaterials. In this framework, different studies have assessed the potential toxicity or biocompatibility of gold nanoparticles, with different sizes, shapes, and surface coatings in zebrafish both in adults and during development.

### 2.1. Toxicity of Gold Nanoparticles in Zebrafish during Development

#### 2.1.1. Uptake and Biodistribution of Gold Nanoparticles

**Figure 1:**
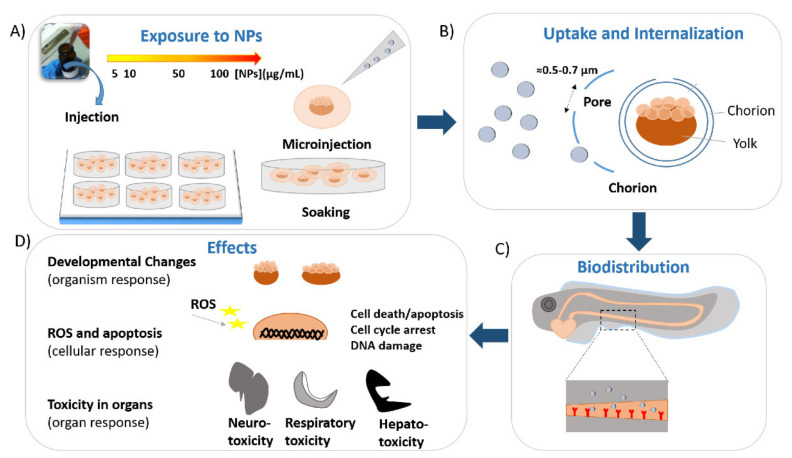
Action mechanisms of nanoparticles on zebrafish. (**A**) Exposure of eggs to nanoparticles dissolved in embryo medium by simple soaking or direct microinjection; (**B**) uptake and internalization of nanoparticles in zebrafish eggs by passive diffusion through the chorion pores (500–700 nm); (**C**) nanoparticles enter the circulatory system and accumulate in different tissues and organs; (**D**) effects of nanoparticles on zebrafish at different levels: organism (developmental changes), cells (ROS generation and apoptosis), and organs (neurotoxicity, respiratory toxicity, and hepatotoxicity). Abbreviation: NPs: nanoparticles; ROS: reactive oxygen species.

Eggs obtained from the spawning of adult wild-type or transgenic zebrafish were collected and rinsed several times with embryo medium (E3 medium). After, eggs were transferred to a plate filled with E3 medium, and the healthy embryos were selected by using a stereomicroscope. Subsequently, embryos at cleavage stages were exposed for a specific temporal window to different concentrations of gold nanoparticles diluted in E3 medium by simply soaking or direct microinjection. Embryos were maintained at constant temperature (27–28 °C) and pH (6.8–7.3). Gold nanoparticles enter the zebrafish eggs by passive diffusion through the pore canals (size around 0.5–0.7 microns in diameter) of their protective barrier, the chorion. They continue their random walk in the embryos and internalize in the inner mass [36,37]. The uptake of gold nanoparticles is fast and sustained from 24 to 48 h, with a stable or increased internalization between the two stages [38,39]. Subsequently, the number of gold nanoparticles internalized in the larvae increases in a dose-dependent manner [40,41], reporting that the gold nanoparticles concentration gradient could be the driving force of their passive diffusion in the eggs [36]. After the internalization, Au-NPs accumulate in different tissues and organs of the embryos and larvae [36,37,42,43]. In particular, by using dark-field optical microscopy and spectroscopy (DFOMS) in real time, the analysis of ultrathin-layer sections (~0.25–4 μm thickness) obtained by cutting embedded larvae incubated for 120 h with chronic doses of gold nanoparticles showed the presence of Au-NPs in different parts of completed growth larva. These include the pectoral fin, swim bladder, notochord, stomach, otic capsule nasal sensory epithelium, brain tissue, and retina [36]. The biodistribution of gold nanoparticles was also analyzed in the case of direct microinjection of NPs in zebrafish embryos at the one-cell stage by surface-enhanced Raman scattering (SERS), as a new tool to observe the nanoparticles distribution, instead of the most common techniques (fluorescence imaging and TEM). First, embryos at the one-cell stage were put in an injection apparatus, and the embryo cell was correctly positioned with respect to the needle for the injection. A small volume (1 nL) of SERS nanoparticles was injected into each egg, and Raman mapping based on SERS was employed to detect the probes in the different zebrafish regions of the embryos and tissues and organs of the larvae. At 6–8 hpf, Au-NPs were mainly localized in the embryo mesodermal region and absent in the yolk [44]. At 14–16 hpf, Au-NPs were detected in the hatching gland, eyes, forebrain, and body musculature. The Au-NP localization, in these early stages of development, suggested that the Au-NPs after the injection extended to various blastomeres and subsequently various tissues and organs. At 20–22 hpf, Au-NPs were present in the embryo dorsal region, while at between 48 and 95 hpf, they localized in the tail, otic vesicle, pharyngeal skeleton, mouth, and brain [44]. Moreover, the biodistribution of different shapes of Au-NPs in zebrafish embryos was analyzed by using a confocal laser-scanning microscope [42]. Zebrafish embryos were exposed to the gold nanoparticles with different shapes between 3 and 5 dpf, considered by the authors to be the temporal window with a higher nanoparticle uptake. Treated embryos and larvae were rinsed in an embryo medium and fixed on a plate with low melting agarose to image the sample. Spherical gold nanoparticles, nanorods, nanourchins, and nanobipyramids were found in the intestinal tract and blood vessels near the eye. Moreover, nanorods also localized specifically in the liver/gallbladder. On the other hand, by coupling two-photon multifocal laser microscopy, with the use of 3 dpf transgenic embryos microinjected with nanobipyramids, it was possible to visualize the tracking of these nanoparticles. Nanobipyramids accumulated in the embryo tail, and several Au-NPs were found free or in clusters in the circulatory systems [42]. Once accumulated in different organs, gold nanoparticles can interact with the biological systems at different levels, leading to toxicity (Figure 1).

The potential toxicity of gold nanoparticles is evaluated by analyzing several toxicological endpoints at different developmental stages, established on both potential organ effects and phenotypical abnormalities. Normally, to obtain a good statistic, three replicate experiments are conducted. The first parameters normally evaluated in a toxicity study are the survival (or mortality) rate and the hatching rate.

After, other toxicological endpoints are also assessed, including the potential presence of malformations, the frequency of movements or swimming activity or touch responses and the heartbeat rate (cardiac activity—heart rate per minute). All these parameters are evaluated in vivo by using a stereomicroscope and at specific growth stages without removing the eggs/larvae from their well. The mortality/survival rate is generally recorded starting from 4 hpf and observed every 24 h, while the hatching process occurs between 48 and 96/120 hpf. The other parameters are evaluated in larvae at 72 of 96 hpf. Furthermore, the effects on the DNA (genotoxicity) and immune system (immunotoxicity) are investigated, as well as organ-specific toxicity (hepatotoxicity, neurotoxicity, and reproductive toxicity).

**Figure 2:**
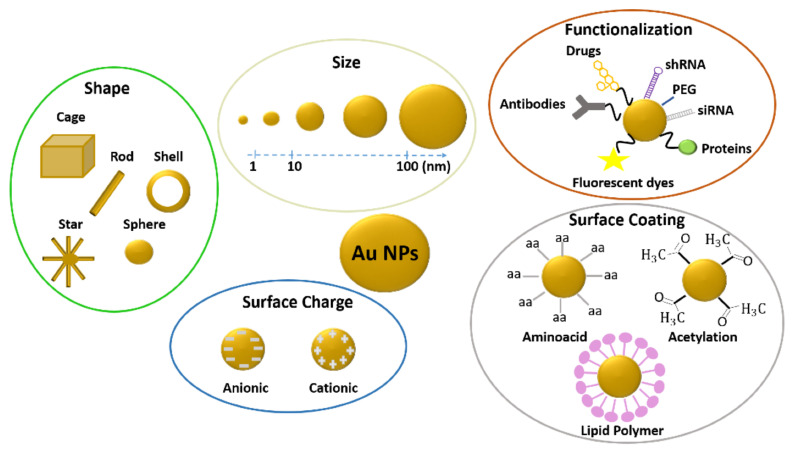
Different factors influencing the gold nanoparticles’ toxicity: shape, size, functionalization, surface charge, and surface coatings. Abbreviations: shRNA: Short hairpin RNA; PEG: Poly (Ethylene Glycol); siRNA: Short interfering RNA.

These adverse effects of nanoparticles depend on several factors, including their concentrations, shapes, size, and coatings as well as their functionalization, stability, and the quality of the medium [45] (Figure 2).

#### 2.1.2. Influence of Gold Nanoparticle Size on Toxicity

The embryos exposure to small (12–35 nm) spherical gold nanoparticles had no significant effects on zebrafish development, with low mortality, no hatching delay, and absence of perturbations on the heartbeat rate for all the tested concentrations [36,40]. After the evaluation of these parameters, the larvae were anesthetized by using an anesthetic with 0.1% phenoxyethanol and observed under a microscope to detect the presence of potential abnormalities. Treated embryos and larvae showed normal development of eyes, otoliths, brain, and tail at 24 hpf and no perturbations in the pigmentation and organs at 48 and 72 hpf [40]. On the other hand, large (86 nm) gold nanoparticles caused a higher mortality rate and a higher percentage of morphological abnormalities in comparison to smaller Au-NPs (12–35 nm), demonstrating a direct and proportional correlation between the toxicity of gold nanoparticles and their size [37]. Moreover, gold nanoparticles resulted in being less toxic to zebrafish during development compared to other similar-sized plasmonic nanoparticles [36,37].

#### 2.1.3. Influence of Gold Nanoparticle Surface Chemistry on Toxicity

Since surface chemistry is considered to be another one of the parameters influencing the toxicity of nanoparticles, different studies have compared the toxicity of unfunctionalized gold nanoparticles with others characterized by the same size and concentration of the latter while using various surface coatings. Bar-Ilan et al. exposed zebrafish embryos to different sizes (3, 10, 50, and 100 nm) of triphenylphosphine monosulfonate (TPPMS) functionalized Au-NPs and to bare colloidal gold nanoparticles (cAu) synthesized by common methods, without extraneous ligand exchange processes [46].

The obtained results showed that TPPMS Au-NPs and cAu caused no toxicity or minimal toxicity for the different tested sizes, with no significant differences between the functionalized and unfunctionalized gold nanoparticles. Moreover, the tested cAu-NPs were found to be less toxic when compared to colloidal bar silver nanoparticles (cAg-NPs) synthesized with the same approach and with the same sizes. Since this difference indicates the possible absence of Au-NP uptake, the authors investigated the internalization of nanoparticles by instrumental neutron activation analysis (INAA). To this end, embryos were euthanized in tricaine (MS-222), rinsed with phosphate buffer solution (PBS), and fixed in paraformaldehyde (4%) prepared in PBS overnight. Embryos were then left to dry. INAA was employed to score the quantity of cAu and cAg accumulated in the different zebrafish tissues. Treated and not treated zebrafish embryos were irradiated for 2 h, obtaining the formation of the two isotopes. The results showed that zebrafish larvae absorbed both cAu and cAg, suggesting that the toxicity of gold nanoparticles was more dependent on their surface coatings than on their size [46]. Other works evaluated the effects of polyvinyl alcohol (PVA)-capped nanoparticles [40,47]. The treatment of zebrafish embryos with bare and PVA-coated NPs did not cause abnormalities in their development.

#### 2.1.4. Influence of Gold Nanoparticle Surface Charge on Toxicity

In this framework, the potential influence of surface charge on the toxicological profile of gold nanoparticles was also investigated. To this end, the toxicity of gold nanoparticles coated with different charged functional groups was evaluated [38,48]. Small gold nanoparticles (0.8, 1.5, and 15 nm) conjugated with positively or negatively charged groups caused adverse effects, while gold nanoparticles with neutral charges did not induce toxicity, confirming the key role of coating chemistry in the gold nanoparticles’ toxicological profile [38,48]. In particular, gold nanoparticles coated with T N, N, N-trimethylammoniumethanethiol (TMAT) (positively charged) induced high mortality, coupled with various morphological abnormalities, whereas Au-NPs coated with the negatively charged mercaptoethane sulfonic acid (MES) induced a high percentage of abnormalities during development with no important lethality in the biological temporal window considered [38,48]. Moreover, both kinds of nanoparticles led to misregulations of genes associated with the immune and inflammation responses and behavioral abnormalities also extended into adulthood [39,48]. Conversely, embryos exposed to Au-NPs coated with neutral 2-(2-(2-mercaptoethoxy) ethoxy) (MEE) and 2-(2-(2-mercaptoethoxy)ethoxy)ethanol (MEEE) did not show any perturbation in the toxicological parameters evaluated [38,39]. Harper et al. supposed that these differences in the toxicity induced by different Au-NPs could be correlated to a different uptake or clearance of the NPs or a different mechanism of interactions. To evaluate this, the clearance and uptake of the different Au-NPs were evaluated by INAA. To this end, zebrafish embryos and larvae were rinsed several times with water to remove all the nanoparticles stuck on the external part of the zebrafish and not internalized. Subsequently, individual embryos were flooded with neutrons to generate radioactive isotopes. The analysis of the radioactive isotopes decay allows for detecting the type and amount of elements that were accumulated in the zebrafish at the beginning.

The embryo’s interaction with positively charged nanoparticles produces different results compared to that interacting with negative and neutral charged nanoparticles. This indicates that TMTA-Au-NPs and MES-Au-NPs operated with opposite mechanisms, considering that TMTA-Au-NPs at a low concentration (2 ppm) induced mortality and were not quickly cleared by the larvae, whereas MES nanoparticles at high doses (50–250 ppm) were not adverse and were quickly eliminated from the larvae [38]. Consequently, the different effects of the three kinds of gold nanoparticles on zebrafish were not related to a difference in the uptake but were due to the presence of different surface coatings. To understand how gold nanoparticles with different coatings were able to cause different effects, a gene expression profile was performed in embryos treated with two groups of nanoparticles (TMAT and MES). RNA was extracted from embryos of 24 and 48 hpf treated with the TMAT-Au-NPs and MES-Au-NPs. Before the extraction, embryos were euthanized with tricaine (MS-222), rinsed with water, and homogenized. Finally, double-stranded cDNA was synthesized and labeled, and the samples were hybridized with a specific gene expression array and were scanned. Important differences in the expressed transcripts were found in embryos of 1 and 2 days post fertilization (dpf), when the surface coating of Au-NPs was directly compared, demonstrating that the surface coatings can affect the gene expression profile [39].

Other research groups subsequently investigated the influence of trimethylammonium ethanethiol or other charged surface groups on gold nanoparticle toxicity. Kim et al. reported that small (1.3 nm) gold nanoparticles coated with the cationic ligand TMAT were toxic to zebrafish, inducing an increase in the mortality rate and incidence of abnormalities [47]. In particular, the exposure to TMAT-Au-NPs caused a clear perturbation in zebrafish eye development. Treated larvae presented gray and smaller eyes with respect to the controls. The expression of different genes implicated in the pigmentation process, apoptosis, and eye embryogenesis were investigated. TMAT-Au-NPs were found to cause the downregulation of genes associated with the formation of the eye and an increase in the apoptotic processes. The tested nanoparticles also induced perturbations in the locomotive behavior of the larvae and abnormal axon development, showing a potential link between neurotoxic effects and behavioral changes [47].

Several studies also showed that ligands employed for therapeutic purposes or to stabilize gold nanoparticles could influence their toxicity [49]. In particular, gold nanoparticles functionalized with mono-sulfonated triphenylphosphine (TPPMS) at the highest concentration tested induced 100% mortality and different morphological defects, including cardiac malformations and peripheral edema. Moreover, zebrafish embryos treated with glutathione (GSH), a ROS scavenger, and TPPM-Au-NPs presented an important reduction of the abnormalities, which suggests that the oxidative stress was the cause of the toxic effect of TPPMS-Au nanoparticles. On the contrary, gold nanoparticles with GSH resulted in being less toxic compared with the TPPPMS-Au-NPs [50]. These studies indicate that coating chemistry, charge, and size are important characteristics that influence gold nanoparticle toxicity in zebrafish during their development. Recently, Ginzburg et al. exposed zebrafish embryos to a mixture of ligand-stabilized gold nanoparticles and Polysorbate 20 (PS20) [51]. Any dose of Polysorbate 20 able to induce an aggregation with the gold nanoparticles caused a significant increase of the mixture toxicity, considering that the PS 20 and Au-NPs alone showed low adverse effects. This finding indicated that the surfactant increased the toxicity of gold nanoparticles. In this framework, Ganeshkumar et al. evaluated the toxicological profile of gold nanoparticles obtained by the sunlight irradiation method. The Au-NPs were functionalized with the ligand folic acid, the dye rhodamine b, or a drug (6-mercaptopurine) (N-Au-NPs-Rd and N-Au-NPs-Mp) [43]. The effects of these nanoparticles were compared to the same unfunctionalized nanoparticles (N-Au-NPs). The use of the latter caused a decrease in the survival rate, a delay in the hatching rate, and a significant incidence of malformations up to a specific concentration. In contrast, N-Au-NPs-Mp caused only a poor pigmentation of the embryos and larvae.

#### 2.1.5. Influence of Gold Nanoparticle Shape on Toxicity

As we have underlined, most of the published studies revealed the effects of sphere-shaped nanoparticles in *D. rerio* during its growth. Gold nanospheres were nontoxic or presented low toxicity, particularly in comparison with other metal NPs. Moreover, large gold nanospheres (86 nm) were found to be more toxic in comparison with the smaller ones (12–35 nm) [36,37]. Since the toxicity of gold nanomaterials is related to their shape, few studies have investigated the impact of gold nanoparticles with other shapes. PEGylated gold nanorods presented concentration-dependent toxicity. Up to a certain dose (20 nM), zebrafish embryos and larvae did not present perturbations in mortality and hatching rates or any relevant malformations [52]. At the highest concentration tested (50 nM), the exposed animals presented increased mortality, a decreased hatching rate, and a severe incidence of defects including fin-fold malformations, tail flexure, yolk sac edema, and pericardial edema. By inductively coupled plasma atomic emission spectroscopy (ICP-AES), the concentration of gold nanorods internalized and accumulated in embryos at 48 hpf and in larvae at 96 hpf was evaluated. The quantity of gold nanorods in the two different stages of development was similar, suggesting that the uptake and biodistribution were widely completed in the first days of treatment. Wang and coworkers compared the impact of cetyltrimethylammonium bromide (CTAB) capped gold nanospheres, nanopolyhedrons, and nanorods on zebrafish. All the tested nanoparticles caused concentration-dependent mortality. Moreover, Au nanospheres induced higher toxicity in comparison to Au nanopolyhedrons and nanorods [53]. Embryos and larvae treated with gold nanoparticles with different shapes were collected, digested with aqua regia, and used to detect their gold content by inductively coupled plasma mass spectroscopy (ICP-MS). The samples treated with GNSs and GNHs presented a higher amount of gold (0.74 µM and 3.3 nM, respectively), in comparison to GNRs (373 µM). On the contrary, in a subsequent study, Patibandla et al. found that gold nanorods stabilized with CTAB and functionalized with polystyrene-sulfate (PSS-GNRs) and with both polystyrene-sulfate and polyallamine hydrochloride (PAH/PSS-GNRs) exhibited the highest toxicity in larvae of 80 hpf, followed by the nanopolyhedrons and the nanospheres [54]. The embryos and larvae treated with nanospheres did not present important changes in the hatching, mortality, and heartbeat rates. On the other hand, animals exposed to gold nanorods showed important perturbations of different toxicological parameters, including a hatching delay, an increase in the mortality rate, and a decrease in the heartbeat rate at the highest concentrations tested. Moreover, the analysis of the expression of genes associated with oxidative stress by using real-time PCR showed a misregulation in treated embryos and larvae [54]. On the other hand, the analysis of the potential apoptosis induced by gold nanorods investigated by using acridine orange (AO) staining of treated embryos showed signs of cell death in all the examined samples. Nanorods coated with PSS and PAH/PSS caused less toxicity in comparison to unfunctionalized GNRs. The different biological behavior of the two groups of nanomaterials was related both to the different shapes of the nanoparticles (spherical and rods) and to surface coating (unfunctionalized versus PSS and PAH/PSS) [54].

Recently, Mesquita and colleagues investigated the effects of CTAB gold nanorods in zebrafish. The toxicity was found to be concentration dependent. At low doses (50 μg/L), no developmental perturbation was detected, while at high doses (114–150 μg/L) embryos were affected by a delay in development (eye and brain) and different morphological abnormalities (tail deformities and pericardial edema, tail elongation, and body size) [41]. These effects were probably due to an induced defect in the cellular process that takes place during the gastrula and segmentation stages. Moreover, the analysis of the genotoxic potential of gold nanorods performed by the Comet assay (single-cell gel electrophoresis assay) on zebrafish embryos treated with subtoxic doses of CTAB-nanorods, showed no DNA damage in zebrafish embryos and larvae between 48 and 96 hpf.

Finally, to assess the toxicity of nanoparticles, it is extremely important to establish the uptake rapidity as well the internal concentration, which produces the incidence of the effects [55]. Moreover, in the case of the effects of metal nanoparticles, the organism can be damaged from both the internalized nanoparticles and the attached fraction. In this framework, the role of the chorion is essential. The chorion protects the embryos up to 48 hpf from the external environment, allowing for the transport of nutrients and gas supply to the eggs [56]. The chorion possesses a negative surface charge [57]. This indicated that the chorion can influence the uptake of metals, and presumably, the internalization will be different for ions and nanoparticles. Truong et al. showed that as ionic concentration decreases, the Au-NP dispersity increases, inducing high mortality, a severe incidence of morphological defects, and perturbations in the larvae behavior [58]. To better understand the interactions between metal ions and metal nanoparticles with zebrafish embryos and larvae, Bohme et al. treated embryos with different nanoparticles, including gold nanoparticles and their corresponding cations [59]. An element-specific internalization of the metal in the different egg regions was observed. Moreover, a different uptake was observed and related to the different dosage forms (ions or nanoparticles). These findings confirm that a different toxicity behavior or ions and correspondent nanoparticles can be due to the different kinetics of uptake in the zebrafish. In this framework, a recent work compared the toxic effects of ionic gold (Au (III)) and small (1.16 and 11.6 nm) gold nanoparticles coated with negatively charged polyvinylpyrrolidone (Au-NPs- PVP) or citrate (Au-NPs CIT) [60]. Au-NPs-PVP and Au-NPs-CIT were less toxic than gold (III) chloride (AuCl_3_). In particular, embryos treated above a middle dose (50 mg/L) of AuCl_3_ presented 100% mortality, while embryos treated with high doses (75 and 100 mg/L) of Au-NPs-CIT and Au-NPs-PVP had low mortality.

All these studies have elucidated that the LC50 for chemically synthesized gold nanoparticles are of 100 ppm or higher, demonstrating that these nanoparticles presented no toxicity or low toxicity in zebrafish during development. Only a few works have assessed the toxicological profile of gold nanoparticles biologically synthesized. In particular, Ramacandra et al. have monitored the effects on zebrafish embryos of 5–50 nm Au-NPs with an anisotropic shape, synthesized using an aqueous extract of *Spinacia oleracea* Linn. These nanoparticles caused concentration-dependent mortality, reaching a value of 100% at the highest concentration tested [61]. Nevertheless, biologically synthesized gold nanoparticles are less toxic than chemical ones.

### 2.2. Toxicity of Gold Nanoparticles in Adult Zebrafish

#### 2.2.1. Effects of Gold Nanoparticles Chemically Synthetized

As we have shown, different studies have investigated and reported the effects of gold nanoparticles on zebrafish during development in waterborne. However, the studies were performed with Au-NPs functionalized with organic compounds and at a dose of up to 250 mg/mL [38,39,40,46,47,48,58], which, according to some researchers, is not the actual concentration of nanoparticles in the environment. To assess possible trigger metabolic perturbations induced by Au-NPs, Geffroy et al. evaluated the effects of low concentrations of food-containing Au-NPs in adult zebrafish [62]. The treatment with food and Au-NPs caused different impairments at the subcellular level, dependent on the exposure time, and gold nanoparticle doses. After a low dose dietary exposure for 36 and 60 days, the expression of different genes involved in DNA repair, apoptosis, and mitochondrial metabolism was evaluated on the control and treated samples. To this end, the RNA was extracted from different organs (brain, digestive tract, liver, or skeletal muscles) of five treated fishes using a specific kit. Subsequently, real-time PCR reactions were used to quantify the expression levels of the single genes. The results showed that mitochondrial perturbations specifically occurred in the muscle and the brain. Moreover, a genotoxicity screening was carried out by means of a modified amplified polymorphism DNA technique (RAPD). This test detected a significant perturbation in the genetic composition. In this framework, Dedeh and coworkers examined the effects of contaminated sediments incorporating small (14 nm) spherical Au-NPs in adult zebrafish for 20 days [63]. In this study, for the first time, as a contamination source for an organism living in a water column, NP-spiked sediment was employed. Gold nanoparticles released in the water column from the sediment caused different adverse impacts. The measurement of acetylcholine esterase activity (AchE) using a simple microplate spectrometer detected variations of brain and muscle AchE. Moreover, DNA damage and variations in genomic expression were observed in treated embryos and larvae [63]. Another study specifically investigated the effects of Ag-NPs of various sizes on the reproductive system, reporting alterations and damage in the ovarian tissue at the histological level [64].

#### 2.2.2. Effects of Gold Nanoparticles Biologically Synthetized

Subsequently, Ramacandra and colleagues reported that the toxicity of adult zebrafish exposed to gold nanoparticles biologically synthesized by extract of *Acalypha indica* was concentration dependent [65]. At low doses (9.7 and 19.4 mg/L), no mortality was observed in the treated adults, whereas at the highest dose tested (58.2 mg/L), the morality reached a value of 100% only after 24 h of treatment. Moreover, aggressive behavior was detected after 12 h of exposure. Once the 50% of lethal concentration (LC50) at 96 h was determined, zebrafish were treated with half of this concentration. The histological analysis of gills and liver tissues showed an absence of damage in the gills but alterations in the liver cells (pyknotic nuclei, membrane damage). Moreover, the analysis of alanine aminotransferase (ALT) levels and aspartate aminotransferase (AST) by colorimetric assay revealed that exposed zebrafish presented a small increase in the levels of these enzymes. The analysis with Schiff’s reagent on microscope slides of zebrafish peripheral blood obtained from the caudal vein showed the absence of genotoxicity. The toxicological profile of these nanoparticles compared with Ag-NPs synthesized with the same approach showed that the gold nanoparticles were less toxic.

As for the zebrafish during development, the toxicological profile of gold nanoparticles with different shapes was also evaluated in adults. In particular, Sangabathuni et al. assessed the biodistribution, clearance, and toxicity of PEGylated and mannose fluorescently tagged Au-NPs with different shapes (stars, rods, and spheres) [66]. After intraperitoneal injection, the tested nanoparticles did not show any toxic effects until 120 h of treatment. By confocal imaging and inductively coupled plasma mass spectrometry, it was shown that the biodistribution of the different gold nanoparticles was dependent both on the mannose coating and the shapes. In particular, gold nanorods were taken up and cleared by the organism very quickly in comparison to the spherical counterpart, while gold stars presented a long and slow uptake and clearance. Thus, the uptake was influenced by the different shapes and the mannose-based interactions.

All the effects of gold nanoparticles with different sizes, shapes, and surface coatings in zebrafish both during both development and adulthood are reported in Table 1.

## 3. Silver Nanoparticles

Currently, silver nanoparticles are increasingly employed in environmental science, electronics, and biotechnology [67,68], thanks to their catalytic and chemical activities, surface-enhanced Raman scattering elements, nonlinear optical behavior, and high electrical and thermal conductivities [69,70]. On the other hand, their well-known antibacterial, anti-inflammatory, and antioxidant [71,72,73,74] properties make them suitable for medical diagnosis and detection and tissue engineering [75]. Moreover, silver nanoparticles are extensively present in daily commercial products, such as toys, textiles, clothing, medical disinfectants, and healthcare products (i.e., cosmetics) [76]. The constant increase in the use of silver nanoparticles results in high environmental and human exposure during production, employment, and disposal [77,78]. Recent studies have elucidated that silver nanoparticles seem to be more toxic in comparison with their bulk form, underlining the importance of assessing their effects on environmental and human health [79]. To this end, several works have assessed the possible interactions of silver nanoparticles with biological systems of different complexities (in vitro and in vivo models), focusing attention on the aquatic organisms, since a high concentration of Ag-NPs is released from used products in surface water and sediments.

### 3.1. Toxicity of Silver Nanoparticles in Zebrafish during Development

#### 3.1.1. Uptake and Biodistribution of Silver Nanoparticles

Different studies have evaluated the impact of silver nanoparticles in zebrafish during their growth [46,61,80,81,82,83,84,85,86,87,88,89,90,91,92,93,94,95,96,97,98,99,100,101,102,103,104,105,106,107,108,109], reporting that they lead to different adverse effects.

As in the case of gold nanoparticles, after spawning wild-type or transgenic fishes, eggs were collected and washed 2/3 times with E3 medium. The healthy eggs were transferred in wells with E3 medium. Embryos at 2/6 h post fertilization were treated for a specific time window with several doses of silver nanoparticles dissolved in E3 medium by simply soaking or microinjection. Embryos were kept at 27–28 °C, in a range of pH 6.8–7.3. Silver nanoparticles of different sizes enter the eggs in few hours by passive diffusion through the pore canals of the chorion. After that, they are caught by the embryo inner mass [37,80,86]. At the different developmental stages, embryos and larvae show a good uptake of silver nanoparticles [80]. Once internalized, Ag-NPs accumulate in different organs and tissues [80,82,87,93,110]. Embryos treated with chronic doses of silver nanoparticles presented Ag-NPs localized in several organs of completed developed larvae, including the tail, brain, retina, heart, and gill arches [80]. In addition, transmission electron microscopy (TEM) images of zebrafish exposed to BSA-capped Ag-NPs showed the presence of well-dispersed or small clumps of Ag-NPs in the heart and brain, with a regular biodistribution in the embryos [82]. In addition, it was shown by histological analysis that silver nanoparticles penetrated the embryos skin and blood tube as aggregated particles [97]. The presence of a high amount of silver nanoparticles in the zebrafish brain during development was subsequently confirmed by quantification of Ag on treated larvae at 96 hpf using inductively coupled plasma mass spectrometry (ICP-MS) [87]. On the other hand, the level of Ag-NPs on the larvae trunk was lower than the brain amount. Moreover, the bioaccumulation of Ag-NPs in 21 dpf larvae indicated two principal target tissues for the Ag-NPs: the liver and the gills [93]. In particular, in the liver, Ag-NPs preferentially accumulated between the gut and liver and in the blood vessel barriers. At the subcellular level, once Ag-NPs enter the cells, they translocated to different cellular organelles. In particular, histological analysis showed that Ag-NPs were mainly distributed in cell nuclei, with only a few NPs in the cellular cytoplasm [82,97]. This specific localization can be correlated to the different genomic damages induced in treated embryos and larvae.

#### 3.1.2. Different Effects of Silver Nanoparticles

Silver nanoparticles induce deleterious effects in exposed embryos, with toxicity strongly concentration dependent [80,85]. Many studies reported that high concentrations of Ag-NPs caused an increase in the mortality rate and a delay in the hatching rate. Moreover, the early growth stages were more sensitive to the treatment, and surviving zebrafish exhibited a severe increase in the incidence of malformations [37,46,61,80,82,84,94,95]. The different abnormalities observed under the stereomicroscope included pericardial edema, bent/twisted notochord, abnormal body axes, tail malformations, small head, absence of eyes or defective eyes, and impaired pectoral fin development with an incapacity to swim [40,46,82]. Few treated larvae were also shorter in length in comparison to the control ones or showed a decrease in touch response [40]. Additionally, larvae exhibited cardiac and circulatory problems (i.e., blood clots and hemorrhages) [46,82,97]. The analysis of the cardiovascular system reported a distorted and thin heart chamber, and, at higher nanoparticle concentrations, atria appeared string like and thin [40]. A relevant abnormality in the heartbeat rate was observed, together with slow pumping of blood and slow pumping efficiency. These phenotypic changes have been correlated to an increase in the oxidative stress that occurs in different tissues and organs, including the liver, intestine, head, and gills [94,97,110]. Increased production of reactive oxygen species (ROS) was detected by incubating exposed larvae of 72 hpf with the cell-permeable CM-H_2_ DCFDA and subsequent observation with a fluorescence microscope. On the other hand, the estimation of total glutathione (TGSH) by the rate of reduction of 5,5–dithiobis(2-nitrobenzoic acid) at 412 nm by GSH in comparison with a standard glutathione curve (colorimetric assay) revealed a reduction of glutathione levels in samples exposed to silver nanoparticles [89]. In agreement with this, different genes associated with the pathway of apoptosis resulted in being misregulated. In particular, the evaluation of the expression of different genes after treatment of embryos with silver nanoparticles by microarray analysis showed that the highest number of genes upregulated (54%) were the ones associated with the apoptosis process (*slc11 a2*, *tp53, dap3*) [90]. Significant perturbations also occurred in the proapoptotic genes (*Bax*, *p21* and *Noxa*), as well as in the ones correlated with the reticulum endoplasmic (ER) stress (*BiP*, *Synv, TNF-α*) and DNA damage [96].

Ag-NPs also produced other specific effects on the zebrafish embryos and larvae.

The specific accumulation and high uptake of silver nanoparticles in the head region suggested that they could induce neurotoxicity. Similarly, the presence of different eye and head defects, such as hypoplastic hindbrain, small head, and eye with reduced pigment, in treated larvae indicated possible perturbations in brain development [87]. For this reason, different works have investigated the possible Ag-NP-induced neurotoxic effects related both to the neurobehavioral profile and gene expression pattern [87,95]. The profiles of various neural development-related genes (*ngn1*, *huC, gfap, NeuroD*) were affected by Ag-NP treatment in embryos analyzed at 24 hpf, indicating that this perturbation could be the cause of head abnormalities of developing organisms [87]. Additionally, treated larvae presented low swimming activity or hyperactivity under different light conditions [95] or low response to touch [92]. In particular, low doses (0.03–3 ppm) of Ag-NPs that did not cause a hatching delay or mortality of abnormalities led to significant behavioral changes [91]. On the other hand, the expression of molecules associated with the cholinergic systems was inhibited [92]. In particular, larvae presented a decrease in acetylcholinesterase (AChE) and propionylcholinesterase (PChE) activities. These alterations, coupled with impaired recruitment of Cd41^+^ cells (T-lymphocytes), indicated that silver nanoparticles also impaired immune system differentiation. The genes related to the immune system were the most affected (18%) after those linked to the apoptosis [90].

Ag-NPs also caused effects on the pigmentation of zebrafish during development by exclusively arresting the differentiation of xanthophores and melanophores. Embryos exposed or injected with Ag-NPs exhibited a reduction of the pigmentation, with a reduction of xanthocytes and melanocytes. Additionally, by using whole-mount in situ hybridization (WISH) detections (with specific synthetized DIG-labelling antisense RNA probes), it was shown that embryos presented a low expression of different xanthophore (*gch2* and *aox5*) and melanophore (*mitfa*, *tyrp1 b*, *oca2*, and *dct*) genes and an increased expression of an iridophore gene (*csf1 b*) [107].

In addition, Ag-NPs affected embryonic hematopoiesis. The gene expression analysis performed with microarray analysis by gene chips on embryos treated with Ag-NPs showed a downregulation of genes in the hemoglobin complex during the embryogenesis and erotogenesis defects, with no perturbation on the mesoderm-hematopoietic commitment and blood vessels [111]. Finally, next-generation sequencing (NGS) analysis of the genome of Ag-NP-treated zebrafish showed strong perturbations in protein synthesis and oxidative phosphorylation [83].

Surprisingly, in the several works analyzed, the LC50 values were quite different [40,80]. This is probably correlated to different physicochemical properties of the studied nanoparticles that influenced the Ag-NP effects. As in the case of gold nanoparticles, the toxicological profiles of silver nanoparticles are also influenced by several factors, including their concentration, size, shape, surface coatings, stability, and medium composition [88,89,112,113,114].

#### 3.1.3. Influence of Silver Nanoparticle Size on Toxicity

For this reason, many studies have defined the physicochemical conditions of silver nanoparticles over toxicological screening and have examined the interaction of different surface-coated and sized Ag-NPs, with controlled particle dispersion [81,95,112]. Zebrafish embryos treated with medium-sized (42 nm) silver nanoparticles exhibited a concentration-dependent mortality, with a value of 100% at the high dose tested (≥ 0.2 nM) and severe malformations. [85,94]. These medium-sized nanoparticles (42 nm) were found to be more toxic than the previously studied small (11 nm) Ag-NPs [36,80], reporting the size-dependent nanotoxicity of silver.

In contrast, research conducted by Mosselhy and coworkers reported that small (9 nm) PVA-coated silver nanoparticles caused a more significant mortality rate and higher delay in the hatching rate in comparison with bigger PVA-capped NPs (30 nm) [105]. This is in agreement with recent work on two sizes (4 and 10 nm) of Ag-NPs [104]. Exposed larvae to the 4 nm Ag-NPs exhibited delayed yolk sac absorption and decreased body length, while those treated with 10 nm Ag-NPs did not present these perturbations. Moreover, the analysis of the expression levels of single mRNA associated with a specific gene of membrane functions and of oxidative stress by means of quantitative PCR (qPCR) revealed an upregulation of the hypoxia-inducible factor, *HIF4,* and *Pxmp2*, a membrane transporter protein, only in embryos treated with 4-nm Ag-NPs.

#### 3.1.4. Effects of Silver Nanoparticle Surface Chemistry and Charge on Toxicity

Subsequent studies have analyzed the effects of size and surface coating simultaneously. To this end, zebrafish were treated with 14 different preparations of silver NPs, including uncapped nanoparticles and nanoparticles stabilized or functionalized with 16-mercaptohexadecanoic acid (Thiol), polyvinylpyrrolidone (PVP), citrate (TSC_1), biocompatible gelatin (BIO), and polymeric coating (Pl) [99]. Two sizes of Ag-NPs were synthesized for each type. Embryos treated with the different silver nanoparticle solutions presented an increase in mortality rate that was dependent both on concentration and nanoparticle size. In particular, the order of toxicity was nanoparticles functionalized with gelatin, functionalized with citrate capper, 16-mercaptohexadecanoic acid capped, and PVP coated. Additionally, heartbeat rate was decreased with the PVP nanoparticles of two sizes, inducing a strong reduction of beat for a minute and reduced mobility in larvae exposed to all the different Ag-NP preparations. These results demonstrated that surface coating possesses an influence on the toxicity of silver nanoparticles. Subsequently, Osborne et al. examined the effect of 10 or 35 nm silver nanoparticle toxicity coated with fulvic acid or citrate on zebrafish [107]. The 10 and 35 nm Ag-NPs induced concentration-dependent mortality and a high incidence of malformations, especially during the early stage of development. The 10 nm Ag-NPs were found to be more toxic in comparison with the bigger ones for all the doses tested (5, 50, 500, 5000, and 25,000 µg/L). On the other hand, the presence of a coating of fulvic acid or citrate strongly decreased the Ag-NPs’ adverse effects. The highest value of mortality rate for 10-nm citrate-coated Ag-NPs was 14% versus a value of 79% for 10 nm bare nanoparticles. Similar results with a size and surface coating influence on toxicological behavior were also reported in the case of 10- or 50-nm PVP-coated Ag-NPs (Ag-NP-PVP) and 10-nm citrate-coated Ag-NPs (Ag-NP-C) [95]. The treatment with 10-nm citrate-coated Ag NPs led to a delay in the hatching rate and a delayed swim bladder inflation, depending on concentration. Neither 10-nm citrate-coated Ag-NPs or 10- or 50-nm PVP-coated Ag-NPs caused perturbations in the mortality rate or incidence of malformations. Consequently, the 50-nm Ag-NP-PVP were found to be more toxic, followed by the 10-nm Ag-NP-PVP and the Ag-NP-C.

Additionally, the effects of the surface charges on the Ag-NPs’ toxicological behavior were assessed in zebrafish during growth. To this end, Lee and colleagues treated zebrafish embryos with various charged-Ag peptide NPs: the negatively charged Ag-CALNNE NPs^−4ζ^ and Ag-CALNNS NPs^−2ζ^ and the positively charged Ag-CALNNK NPs^+ζ^. Nanoparticles coated with all three peptides caused a concentration-dependent toxicity in terms of incidence of malformations, including pericardial edema, yolk sac edema, spinal cord and tail flexure, and dissymmetric or small eyes. The three charged -Ag peptide NPs presented different degrees of toxicity. Ag-CALNNE NPs^−4ζ^ were the more toxic, while the Ag-CALNNK NPs^+ζ^ were the most biocompatible nanoparticles. Moreover, comparing these effects with citrate-capped Ag-NPs of the same size previously studied, it was shown that Ag-CALNNE NPs^−4ζ^ and those positively charged were, respectively, more biocompatible and equally biocompatible in comparison with their citrate-coated counterparts. On the other hand, the low negatively charged nanoparticles presented lower toxicity compared with the citrate-coated NPs. These findings clearly showed that Ag-NP toxicity is strongly dependent on surface coating charges [86].

Some studies have examined the roles of other potential factors, including the presence of sediment and aquatic plants, the composition of the exposure medium, and biogenicity, on Ag-NP toxicity.

#### 3.1.5. Influence of Sediment, Aquatic Plants, and Medium Composition on Silver Nanoparticle Toxicity

The role of sediment and aquatic plants in Ag-NP behavior was examined by treating the embryos with silver nanoparticles possessing coatings such as polyvinylpyrollidone (PVP-Ag-NPs) or gum arabic (GA-Ag-NPs) diluted in different microcosm matrices (W: water only; WS: water plus sediment; WP: water + plants; WPS: water + plant + sediment) [106]. The mortality rate of zebrafish treated with GA-Ag NPs in three different matrices, WP, WPS, and WS, presented lower values with respect to the those exposed to GA-Ag-NPs in water. The same was also observed for the PVP-Ag-NPs even if the difference between matrices was less significant. These findings indicated that plants alone or with sediments seem to be protective against GA and PVP-Ag-NP toxicity. Sediment alone reduced the adverse effects only in the case of PVP-Ag-NPs. These results demonstrated that the nature of the environmental matrix exerted an influence on the toxicological behavior of silver nanoparticles. On the other hand, the effect of biogenicity was tested by Verma et al., evaluating the effects of silver nanoparticles synthetized using two Gram-negative (*E. coli*, ECAg-NP and *Salmonella typhimurium*, STAg-NP) and two Gram-positive (*B. thuringiensis*, BTAg-NP and *S. aureus*, SAAg-NP) bacteria [103]. The mortality rate was influenced by the biogenicity and dose of nanoparticles, while the hatching and heartbeat rates decreased in a dose-dependent manner. Up to a certain dose, all the different nanoparticles cause significant effects. To facilitate cell death detection on treated samples, zebrafish embryos were stained with acridine orange (AO) and observed with a fluorescence microscope. SAAg-NP and BTAg-NP induced less important malformations in comparison with ECAg-NP and STAg-NP. On the contrary, ECAg-NP and STAg-NP caused higher apoptosis (higher AO signal) in the tail and head area, with respect to the other two kinds of nanoparticles. These findings demonstrated that the toxicity of the different nanoparticles was influenced by their concentration, biogenicity, and exposure time.

The effects of the medium composition were assessed on embryos treated with PVP or citrated-coated Ag-NPs of 20 or 100 nm dissolved in different media (embryo medium, CaCl_2_, and ultrapure water) [81]. The fish development was normal in embryo medium, while the one in CaCl_2_ and ultrapure water led to high adverse effects, with 100% mortality for 20 nm NPs at 120 hpf. Additionally, smaller Ag-NPs (20 nm) were acutely toxic in comparison to the biggest ones (100 nm), and PVP-capped nanoparticles were found to be more toxic than those coated with citrate and with the same dimension. The influence of surface coating and size on NP toxicity is clear.

#### 3.1.6. Influence of Silver Dissolution on Toxicity

However, the majority of silver nanoparticles go through dissolution in aquatic environments, and numerous results reported in the literature are related to silver ions release [102]. Many studies have been performed to examine the contributions of silver ions and silver nanoparticles on toxicity, even if the obtained results were in contrast. In this framework, Wang and coworkers exposed zebrafish embryos to bare Ag-NPs (Bare-nAg), PVP Ag-NPs (PVP nAg), and Ag-NPs with a dispersant (DIS- Ag-NPs) [108]. Adverse effects were found to decrease in the order of DIS-nAg > PVP-nAg > Bare-nAg. Considering that bare nanoparticles and DIS-Ag-NPs had, respectively, the lowest and highest amount of silver ions, it is clear that silver ions need to be considered in nanosilver toxicity. Similarly, the analysis of the molecular mechanism of the adverse effects of silver nanoparticles in zebrafish reported that Ag-NP toxicity was correlated with the soluble fraction of the silver [83]. The gene expression of embryos exposed to the different preparations presented important alterations, with a more pronounced impact in the case of silver ions. In particular, genes linked with protein synthesis and oxidative phosphorylation were the most affected, with a similar perturbation in the case of Ag-NPs and Ag^+^, suggesting that the different silver states presented a comparable toxicity mechanism. In particular, after 24 h of treatment, zebrafish had a misregulation of the phosphorylation, with a fast restoration (after other 24 h). With a similar trend, zebrafish exposed to Ag^+^ for 24 h showed a reduction in oxygen consumption with subsequent recovery. A similar correlation was found when comparing the impact induced by silver ions with that induced by equivalent doses of carbonate-coated silver nanoparticles with the same amount of silver contained in the ionic form. Zebrafish were placed in an embryo medium with a low content of chloride [98]. Moreover, the Ag-NP solution was treated with a silver chelating agent, cysteine, to erase the silver ionic form. The magnitude of effects induced by the Ag-NPs and Ag^+^ were similar to each other and both correlated to the stage of development. Moreover, they were mitigated or erased by the addition of cysteine in the medium. Finally, by analyzing and comparing the results from other studies [40,46,80,82,85,90,95,99,107], it was clearly shown that the adverse effects of Ag-NPs were strongly dependent on silver ions.

In contrast, Cambier et al. demonstrated that silver nanoparticles and soluble Ag possess similar targets [93]. The different results obtained in these studies can be related to the different experimental protocols used (e.g., life stage analyzed), differences between the nanoparticles themselves (e.g., surface coating and size), or their behavior (e.g., dissolution and agglomeration). To verify this hypothesis, Bole and colleagues carried out a test and comparison of the behavior of Ag-NPs and Ag NO_3_ in zebrafish by using an accurate experiment design [102]. Eggs were treated with Ag-NPs and Ag NO_3_ in four different screenings between 24 and 120 hpf for 24 h. Embryos and larvae at different hpf were found to be more sensitive to Ag NO_3_ compared to Ag-NPs (difference of 200 times). Moreover, the zebrafish sensitivity to the nanomaterials increased with the stage of development, with a similar LC_50_ at the different developmental time points for Ag NO_3_ and Ag-NPs, suggesting similar targets for both. Additionally, Ag-NPs and Ag NO_3_ caused a strong inhibition of Na^+^ uptake, with a similar magnitude of effects. These results showed that zebrafish were less sensitive to Ag-NPs than Ag NO_3_, as already reported by previous works [107,108].

Finally, a recent work assessed the effects on zebrafish during the development of leachates released by Ag-NP-coated socks (sock-Ag-NP) and of centrifugate (spun-Ag-NP) free of Ag-NPs [94] in comparison with the that induced by Ag NO_3_. Embryos treated with the highest dose of sock-Ag-NP and spun-Ag-NP (0.83 mg/L) preparations presented a mortality of 100% after the first 24 h. Moreover, embryos and larvae treated with lower doses (0.05 mg/L) had a delay in hatching rate, different malformations, and an important upregulation of *sod* mRNA levels in the case of spun-Ag-NPs. On the contrary, embryos treated with Ag NO_3_ presented a high LC_50_ (0.80 mg/L) and no malformations, indicating that both sock-derived solutions exerted higher toxicity in comparison to the Ag NO_3._ This is in contrast with previous findings that reported the lower toxicity of silver nanoparticles in comparison to the ionic form [46,82,89,112]. However, the discrepancy could be attributed to the different Ag-NP origins, indicating that the effects of Ag-NPs derived by commercial products can be dependent on other components included in the production process.

### 3.2. Toxicity of Silver Nanoparticles in Adult Zebrafish

Different studies have also deeply investigated the possible impact of Ag-NPs in adult zebrafish. This is a crucial point since the adults in ecosystems represent the principal origin for bioaccumulation leading to the biomagnification of xenobiotics [115]. Additionally, the behavior and interactions of adult zebrafish can be different from those reported during development.

Silver nanoparticles were found to be toxic to adult zebrafish in most of the cases. Griffit et al. estimated an LC50 of 7.07 mg/L [100]. The main route of exposure, internalization, and toxicity of Ag-NPs in adult zebrafish is represented by the gills [116]. By this route, Ag-NPs accumulate in different organs. The different toxicological studies have mainly examined the organ-specific toxicity induced by silver nanoparticles in adult organisms.

**Figure 3:**
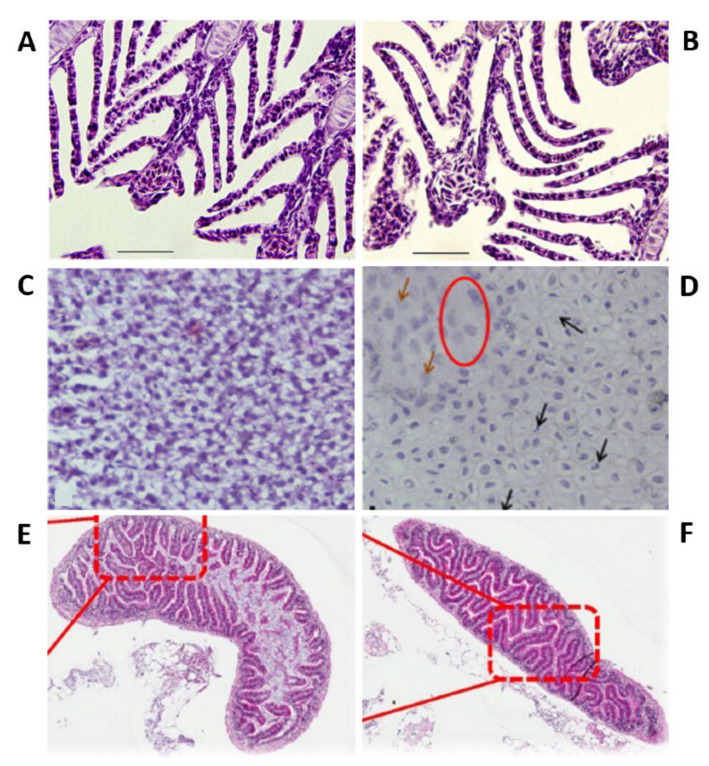
Images of adult zebrafish: gills from (**A**) control sample and (**B**) treated with Ag-NPs, liver from control (**C**) control sample and (**D**) treated with Ag-NPs, and intestine (**E**) control sample and (**F**) treated with Ag-NPs. Reproduced with permissions from Devi, Aquatic Toxicology; published by Elsevier, 2015; Griffitt, Aquatic Toxicology; published by Elsevier, 2013; Osborne, ACS Nano; published by American Chemical Society 2015.

As the principal route of Ag-NP exposure, the gills represented the first organs to be impaired by Ag-NPs. Prolonged and chronic exposure to Ag-NPs leads to their accumulation in the gills in a concentration-dependent manner [117,118]. Moreover, microarray analysis showed that the treatment induced concentration-dependent perturbations in the expression of several genes that are predominantly linked with morphogenesis, developmental mechanisms (i.e., skeletal and organ development and cell growth and migration), and DNA repair and damage [117]. Moreover, both intracellular and intracellular processes were perturbed. These results indicated that chronic Ag-NP treatment had an impact both outside and inside the cell and that it induced DNA damage with probable ROS production. Additionally, Ag-NP internalization in the gills affected ionoregulatory functions, with a perturbation in all the ions’ flux and, in particular, a reduction in the activity of gill Na^+^/K^+^-ATPase effecting the osmotic balance (Figure 3A,B) [83,112,117,119]. Additionally, treated zebrafish presented high levels of cortisol and glucose and low levels of electrolytes in the plasma. The increase of cortisol indicated a condition of stress in the treated zebrafish [119]. Additionally, evident signs of respiratory toxicity were reported in the case of adults treated with chemically produced NPs [100] and with two different kinds of biologically synthesized Ag-NPs (extract of *Acalypha indica* and *Malva crispa* Linn.) affected by a significant increase in the respiratory rate [65,120].

Ag-NPs also enter the adult by ingestion with food, leading to bioaccumulation in the liver and consequent perturbations in this organ and the gut [96]. The hepatotoxicity caused in adult zebrafish by silver nanoparticles was carefully evaluated to examine the roles of apoptosis and oxidative stress in adult zebrafish treated with silver nanoparticles [110]. The histological analysis of the paraffin sections of liver of treated adults marked with hematoxylin and eosin of treated adults revealed several morphological alterations and apoptotic signs (pyknotic nuclei) (Figure 3C,D). On the other hand, the mRNA expression of Metallothionein 2 (MT2) increased depending on the tested doses, suggesting the presence of silver ions in the liver. Additionally, the lipid peroxidation of the tissues, evaluated by measuring the amount of hepatic MDA, a product of lipid peroxidation, and the total GSH, of exposed zebrafish were increased, indicating, respectively, that silver nanoparticle exposure caused oxyradical production and that the liver reacted in defense to this production. The reduction of antioxidant enzyme expression (oxyradical-scavenging enzymes catalase, Cat, and glutathione peroxidase 1 a, GPx1 a), evaluated by a real-time reverse transcription-polymerase chain reaction (RT-PCR), suggested the increase of oxyradicals and hydrogen peroxide with consequent oxidative stress. Moreover, the detection carried out by using γ-H2 AX, a phosphorylated form of H2 AX, showed that Ag-NPs induced DNA damage in the liver. Furthermore, real-time RT-PCR also revealed an upregulation of apoptotic genes (*Noxa* and *Bax*). These results reported that Ag-NP treatment caused hepatotoxicity with DNA damage, apoptosis, and oxidative stress. It was subsequently reported that these effects can be partially reduced by sulfidation [115]. Additionally, the gut of exposed zebrafish presented important necrosis in the villi, without histopathological changes [118]. Moreover, the treated adults had a high level of metallothionein 1 (MT1) [118].

In another study, Ma and colleagues evaluated the effects of Ag-NPs on the gut microbiota of adult male and female zebrafish [121]. Surprisingly, a different impact was observed in the two sexes. The composition of bacterial species of the gut of the male fish was strongly altered in terms of richness and diversity, while that of the females did not present evident changes. In male fish, the abundance of *Fusobacteria* and the genus *Cetobacterium* was increased, while that of *Proteobacteria* was decreased. The same research group also explored the effects of Ag-NPs on the fecundity on adult zebrafish [122]. The fecundity was evaluated by counting the average amount of embryos produced every day by each single female.

Zebrafish exhibited a reduction in reproductivity. A significant increase of reactive species production (ROS) in both testicular and ovarian tissues was probed by dichlorofluorescein diacetate after silver nanoparticle exposure, coupled with an increase of apoptotic cells, detected by TUNEL analysis in both tissues. The transcription level of several genes related to the apoptosis mediated by mitochondria (*bax*, *bcl-2*, *caspase-3,* and *caspase-9*) showed an alteration in the profile of all the analyzed genes. The impact of Ag-NPs on fecundity was also investigated for small (5 nm) Ag-NPs coated with PVP/PEI. In this case, the growth of embryos obtained by the spawning of the treated zebrafish was also observed [101]. After 14 days of exposure, the adult zebrafish exhibited a reduction in reproduction and a strong vacuolization of the liver, while the resulting embryos were affected by a high incidence of abnormalities. Finally, after Ag-NP injection by the intramuscular route, signs of toxicity with tissue damage were also observed in the kidneys, spleen, and gallbladder, coupled with upregulation of tumor necrosis factor alfa (TNFα) and proinflammatory cytokine interleukin 1 (IL1) in all these organs [123].

#### 3.2.1. Influence of Silver Nanoparticle Surface Chemistry on Toxicity

Additionally, as for the zebrafish embryos and larvae studies, the influence of surface coating and size on Ag-NP toxicity was investigated [100,105,107,108,110,112]. Zebrafish were exposed to polyvinylpyrrolidone (PVP)-coated silver nanoparticles (PVP-Ag-NPs) [112] and silver ions. The nanoparticles resulted in being toxic in a very short temporal window. All the fishes treated with the highest concentration (143 μg/L) of nanoparticles died within 24 h of exposure. The anterior ventral surface of their body was characterized by blood extravasation. Moreover, after 30 min of treatment, the animals started to show the first evidence of stress, with an increased rate of respiration and operculum movement, suggesting respiratory toxicity. Subsequently, fishes lost equilibrium and died. On the other hand, silver ions present a concentration-dependent mortality, with no mortality at the highest concentrations after 24 h, and stress signs similar to those detected in the case of adult zebrafish treated with silver nanoparticles. The toxicity of Ag-NPs was attributed to the nanoparticles themselves and not to the coating’s presence, and silver ions were also toxic for adult zebrafish. The found value of LC50_48 h_ was different from that of metal oxide-coated Ag-NPs [100] and also from the LC50_24 h_ of smaller nanoparticles (5–20 nm) [110]. On the other hand, the possible influence of Ag-NP size was examined on target organs (gills and intestines) (Figure 3E,F). To this end, adult zebrafish were treated with two sizes of citrate-coated nanoparticles for different exposure times [124]. Fish exposed to the smallest Ag-NPs (20 nm) presented morphological changes in the gills coupled with inflammation and important hyperplasia, while the ones exposed to bigger (110 nm) Ag-NPs showed the same type of perturbation but less significant. On the contrary, 110-nm Ag-NPs caused more prominent damage in the intestinal structure in comparison with 20-nm NPs. Moreover, 20-nm Ag-NPs caused a higher reduction of the gill and intestine ATPase activity versus bigger NPs.

#### 3.2.2 Influence of Silver Nanoparticle Dissolution on Toxicity

As we have already discussed for development, since in an aquatic environment, silver nanoparticles release silver ions [125], it is important to discriminate the adverse effects due to the Ag-NPs from those due to silver ions. For this reason, Griffit et al. exposed adults and juvenile fish to silver nanoparticles and evaluated the particle dissolution and concentration before and during the treatment. Before the exposure, the dissolution was low. Ag-NP solution was toxic to zebrafish with an estimated LC50 of 7.07 and 7.2 mg/L for adult and juvenile zebrafish, respectively [100]. The analysis of the silver nanoparticle solutions after the treatment showed that > 90% of the nanoparticulate was eliminated within 48 h from the water column, indicating that the toxic effects were probably not due to the particle dissolution. The same authors explored the interaction of the composition and dissolution of nanoparticles in adult gills after treatment with toxic silver and copper nanoparticles and their soluble counterparts [116]. The work aimed to understand if the interaction of the gills to the nanoparticles was correlated with the nanoparticle composition and if this was driven by the dissolution. The levels of Ag associated with the gills were found to be higher after exposure to silver nanoparticles than treatment with soluble silver, indicating that the gill burden of silver received a contribution from the nanoparticles themselves. Moreover, the effects on gill histology were different between the silver nanoparticles and soluble form (nitrate silver) exposure. Female zebrafish treated with silver nanoparticles presented no changes in gill filament morphology, while those treated with the soluble form presented toxicity, with a strong thickening of the filament and with an inhibition of Na^+^/K^+^-ATPase activity. This difference was unexpected since both nanoparticles and soluble exposure presented the same amount of soluble silver. Moreover, the analysis of gene patterns associated with proliferation, apoptosis, and mitogenesis showed a different expression for the nanoparticles and the soluble form, indicating that each treatment induced an impact by a different mechanism. These results reported that even if the amount of soluble silver is the same, the biointeraction of both nanoparticles and soluble exposure changes depending on the ion release rate.

#### 3.2.3 Effects of Silver Nanoparticles Biologically Synthetized

Additionally, in the case of adult zebrafish, most of the reported studies have assessed the possible toxicity of silver nanoparticles chemically synthesized. Only a few works have shown the impact of biologically synthesized Ag-NPs in zebrafish versus the chemically produced ones. For the first time, Ramachandra et al. treated adult zebrafish with biologically synthesized silver nanoparticles obtained from the aqueous extract *Spinacia oleracea* Linn., reporting a concentration-dependent toxicity. No mortality was detected at low doses (15 μg/L), while a 100% mortality rate was found at the highest dose tested (31 μg/L). The animals presented aggressive behavior after only 6 h of treatment, and extravasations of blood in the ventral surface of the body were present. Cytological analysis revealed morphological abnormalities in the liver tissue and cells of the treated adults. Moreover, the zebrafish presented immunotoxicity and genotoxicity [61]. The same research group has also analyzed the behavior of Ag-NPs synthesized by an extract of *Acalypha indica* [65]. Ag-NPs were found to be strongly toxic to zebrafish with 100% mortality after only 12 h of treatment with the highest concentration tested. Fish presented aggressive behavior. Once the LC50_96 h_ was determined, zebrafish were exposed to half the value of this concentration. The treatment led to immunotoxicity and oxidative stress in adult organisms. In particular, Ag-NPs caused histological changes in gills and liver tissue, (pyknotic nuclei and membrane damages), a high level of reactive oxygen species in liver cells, increased levels of liver enzymes, and nuclear abnormalities in blood cells [65]. Moreover, the mRNA expression of genes associated with immune response and oxidative stress was misregulated. These nanoparticles were found to be more toxic than gold nanoparticles synthetized with the same procedure. Concurrently, another study by Krishnaraj and coworkers treated zebrafish with silver nanoparticles by using an aqueous extract of a medicinal herb, *Malva crispa* Linn. [120]. These nanoparticles were extremely toxic to zebrafish, with an L50_96 h_ concentration of 142.2 µg/L. Zebrafish presented signs of stress after only 6 h of treatment. Subsequently, adult zebrafish were exposed to half of the LC50_96 h_ value used to investigate the toxicity mechanisms. The treatment led to clear oxidative and genotoxic effects. In particular, oxidative stress caused morphological changes in the liver and gill (irregular cell outlines, pyknotic nuclei) tissues, while genotoxicity led to a loss in cell contact in liver parenchyma cells and nuclear abnormalities (lobed nuclei, blebbed nuclei) in peripheral blood cells. The oxidative stress and the genotoxicity were confirmed by the analysis of genes related to immune (TLR22, TLR4, NFKB, IL1 B, CEBP, TR) and stress (MTF-1, HSP70) responses [120], with their up- or downregulation. The toxicity of these biologically synthetized Ag-NPs was lower than that of the chemically synthetized nanoparticles. Finally, Sakar et al. compared the in vivo toxicity of biologically and chemically synthetized Ag-NPs. The biological ones were obtained by using a leaf extract of Guava (*Psidium guajava*). The L50_96 h_ concentration was lower for biologically synthetized Ag-NPs, reporting their lower toxicity in comparison to their chemical counterpart [113]. Moreover, both kinds of nanoparticles induced damage in the ovarian tissue, with maximum alterations in the case of chemically synthesized NPs (follicular dystrophy and atresia) and a medium effect in the case of biologically synthetized Ag-NPs (mild atresia). The results of the various studies on the biologically produced Ag-NPs were in agreement, even if the values of LC50_96 h_ concentrations determined by Sakar and Krishnaraj were different. This could be attributed to the different types of plant extract employed for the synthesis process. On the other hand, the lower toxicity of biologically synthetized Ag-NPs versus their chemical counterparts is probably related to the presence of biomolecules on their surface [65].

All the effects of silver nanoparticles with different sizes and surface coatings in zebrafish both during both development and adulthood are reported in Table 2.

## 4. Conclusions

The use of plasmonic nanoparticles in different fields is constantly growing thanks to their outstanding properties. However, the potential risks of these nanomaterials for the ecosystem and human health remain a great concern. The present review focuses on the potential effects of the most commonly used plasmonic nanoparticles, gold and silver, in aquatic organisms. We integrated the findings of many studies, reporting a different toxicological profile for gold and silver nanoparticles in zebrafish both during development and in adulthood. These profiles are both dependent on various critical factors, including chemical composition, concentration, surface coating (chemistry and charge), size, stability/aggregation, and medium composition and quality. The effects of Au-NPs are also affected by the particle shape, while those of Ag-NPs are affected by the Ag^+^ dissolution. Both kinds of nanoparticles penetrate the embryos through the chorion pores, and in the adults, by the gills and ingestion, finally accumulating in specific tissues and organs. However, the degree of toxicity of silver and gold nanoparticles differs, even if evaluated in the same biological system. In particular, gold nanoparticles result in inducing minimal or low toxicity, characterized by small changes in the different biological parameters examined in embryos and larvae. Adults present signs of genotoxicity and damage at the histological level in the ovary and liver. On the other hand, silver nanoparticles caused strong developmental toxicity, with perturbations in various toxicological endpoints, including mortality and hatching rates, incidence of abnormalities, heartbeat rate, and swimming activity or touch response. Several researchers reported widespread organ-specific toxicity in the treated adults, mainly correlated with oxidative stress. In particular, Ag-NPs induce effects in the gills accompanied by respiratory toxicity and in the liver by morphological alterations. It has been also observed that the Ag-NPs affected the reproductive system, leading to a fecundity reduction. These specific impacts are correlated with the misregulation of various genes involved in different processes, which are carefully summarized and discussed. We believe that this overview of the current knowledge on the toxicological behavior of gold and silver nanoparticles will be of great importance in view of their future applications.

## Figures and Tables

**Table 1:** Effects of gold nanoparticles with different sizes, shapes, and surface coatings in zebrafish.

Life Stage	Shape	Size	Surface Coatings	Concentration and Time of Exposure	Mortality Value vs. Dosesor LC50	Results	Ref.
Embryos	Sphere	12 nm	Citrate	0.025, 0.05, 0.10, 0.20, 0.40, 0.60, 0.80, 1.0, 1.2 nM, from cleavage-stage to 120 hpf	31% at 1.2 nM	Effects stochastic dependent from concentration—low mortality—different malformations (fin fold abnormality, tail and spinal cord flexure and truncation, cardiac malformation, yolk sac edema, and acephaly).	[36]
Embryos	Ellipse	86 nm	Citrate	0–78 µg/mL from 0.75/2.25 to 120 hpf	3% at 78 µg/mL	Normal development—effects not concentration dependent—larger Au-NPs (86 nm) more biocompatible than smaller (12–35 nm).	[37]
Embryos	Sphere	0.8, 1.5, and 15 nm	TMAT MES MEE MEEE	0.016, 0.08, 0.4, 2, 10, 50 and 250 mg/L, from 2 to 120 hpf (5 days)	- 100% at 250 ppm TMAT- Ag-NPs - 55–60% at 250 ppm MES-Au-NPs - <10% at 250 ppm MEE-Au-NPs - <10% at 250 ppm MEEE-Au-NPs	TMAT-Au-NPs: increased mortality—negligible malformations. MES-Au-NPs: no significant lethality—increased incidence of abnormalities. MEE-Au-NPs and MEEE-Au-NPs: no adverse effects.	[38]
Embryos	Sphere	1.5 nm	TMAT MES MEEE	0.016, 0.08, 0.4, 2, 10, 50 and 250 ppm, from 6 to 48 hpf	- 100% at 10 ppm TMAT- Au-NPs - 40% at 10 ppm MES—Au-NPs	TMAT-Au-NPs: high mortality—misregulations of genes associated with immune and inflammation responses. MES-Au-NPs: high percentages of abnormalities—misregulations of genes associated with immune and inflammation responses. MEE-Au-NPs: no biological responses.	[39]
Embryos	Sphere and oval	15–35 nm	PVA	10, 25, 50, 75, and 100 mg/mL, from 8-cell stage to 72 hpf	≤3 at 100 mg/mL	No increase in mortality—no hatching delay—no defects in the development—no effects on the heartbeat rate—no perturbations in the touch response for all the tested concentrations.	[40]
Embryos	Rod	20 nm × 7 nm	CTAB	50, 60, 72, 87, 104, 125, and 150 μg/L, from 6 to 96 hpf	L50_96 h_= 110.2 μg/L	Toxicity concentration dependent—at low doses (50 and 87 μg/L) no developmental perturbation—at high doses (125 and 150 μg/L) delay in the development (eye and brain) and different morphological abnormalities (tail deformities and pericardial edema, tail elongation, and body size)—no DNA damage.	[41]
Embryos	Sphere Rod Urchin Bipyramid	79 79 78 5014	- PVP - PEG	5 mg/L, from 3 to 5 dpf	-	Spheres: reduced amount of the neutrophils. Rods and urchins: no effects on the immune system—increased total swimming distance. Bipyramids: no effect on immune systems—under stress-induced, reduction of the swimming distance.	[42]
Embryos	Sphere	33–346 nm	Folic acid	0.325, 0.65, 0.97, 1.3, 1.62, 1.95, 2.27 and 2.6 ng/100 µL, from 2 hpf to 96 hpf	-	Toxicity concentration-dependent—decrease of survival rate—delay in the hatching rate—presence of different malformations (length of larvae and poor pigmentation).	[43]
Embryos	Sphere	25 and 40 nm	Citrate	Microinjection at one-cell stage with 1 nL (5 × 10^−18^ mole)	-	Normal development of somites—normal response to touch at 4 dpf—normal function of the cardiovascular system and other organs—normal expression of different genes (*ntl*, *gsc*, myoD, and β-globin).	[44]
Embryos	Sphere	3, 10, 50, and 100 nm	Bare TPPMS	50, 5, 0.5 and 0.05 mg/L, from 4 to 120 hpf	cAu3 negligible cAu10 negligible cAu 50 negligible cAu 100 negligible	No increase in mortality—no significant morphological defects—no differences in effects between unfunctionalized and functionalized NPs.	[46]
Embryos	Sphere	1.3 nm	TMAT	0.08, 0.4, 2, 10 and 50 mg/L, from 4 hpf to 120 hpf	LC50_120 hpf_ =30 mg/L	High mortality rate and high incidence of abnormalities—perturbations in the eye development, with grey and small eye—downregulation of genes involved in the eye formation and apoptosis processes—abnormal axon development.	[47]
Embryos/adults	Sphere	1.5 nm	TMAT MES MEEE	50 mg/L, embryos from 6 to 120 hpf, adult from 4 hpf to 122 days	20% TMAT-Au-NPs 50% MES-Au-NPs	MES and TMATAu NPs: Embryos: hypo-locomotor activity. Adults: low survivorship into adulthood and abnormal behavior. MEEEAu NPs: Embryos: normal locomotor activity.	[48]
Embryos	Sphere	1, 2.8, 3.1, 3.6, and 3.9 nm	MEEE	2.3, 5, 10.7, 23.2, 50 µg/mL, from 6 hpf to 5 dpf	88% at 50 µg/mL Au-NPs + 0.003 PS20Lily Zhao 100% at 23.2 µg/mL Au-NPs + 0.3 PS20	Au-NPs and PS20 alone have low toxicity—mixtures of Au-NPs and PS20 presented increased toxicity.	[51]
Embryos	Rod	48 × 16 nm and 51 × 13 nm	CTAB	1, 5, 10, 20 50 nM, from 4 to 120 hpf	-	Toxicity concentration dependent—up to middle dose, no perturbation in mortality and hatching rates and incidence of malformations—at the highest dose tested increased mortality, decreased hatching rate and presence of severe malformations (pericardial edema, yolk sac edema, tail flexure, and fin-fold abnormality).	[52]
Embryos	Sphere Rod Polyhedron	46 nm 76 × 23 nm 38 nm	CTAB	0–15.7 µM, from 4 to 80 hpf	LC50_80 hpf_ Sphere: 0.11 nM Rod: 1.54 µM Polyhedron: 0.13 µM	Mortality and hatching rates concentration dependent—order of induced mortality: Au nanospheres > nanopolyhedrons > nanorods—different malformations (several yolk sac edema, cardiac edema, bleeding, skeletal defects, lack of pigmentation, and tail/spinal cord flexure) with the highest percentages induced by polyhedrons.	[53]
Embryos	Sphere Rod	38.1 nm × 12 nm × 52 nm	Sphere: PSS Rods: PSS PAH/PSS	0.01, 0.025, 0.05 and 0.1 nM, from 2 to 72 hpf	-	Spheres: no important changes in hatching, mortality, or heartbeat rates. Rods: increased mortality—decreased hatching and heartbeat rates—perturbations in the expression of oxidative stress genes. Different shapes of nanoparticles and surface coatings affected the toxicity.	[54]
Embryos	Sphere	1.2 nm	MPA	0.08, 0.4, 2, 10 and 50 mg/L, from 6 to 120 hpf	<13% at 50 mg/mL	The ionic concentration of the EM influenced the toxicological profile—low mortality and percentage of malformations at highest ionic concentrations tested—normal motor activity.	[58]
Embryos	Sphere	1.16 and 11.6 nm	Citrate PVP	0, 1, 12.5, 25,50, 75, and 100 mg/L, from 4 to 96 hpf	LC50 above 100 mg/mL	Low mortality at the highest concentration of Au-NPs-CIT and Au-NPs-PVP tested—no hatching rate delay—no significant incidence of malformations.	[60]
Embryos	Sphere	5–50 nm	-	100–300 mg/mL, from 4 to 96 hpf	100% at 300 mg/mL	100% mortality rate (concentration dependent) at the highest concentration tested—one abnormality (tail malformations).	[61]
Adults	Sphere	12 and 50 nm	Citrate	0.04 and 0.1 mg/day/g fish body weight, 36 or 60 days	No mortality	No mortality—different impairment at the subcellular level, dependent on the exposure time and Au-NPs sizes and doses—mitochondrial perturbations in the muscle and the brain—perturbations in the expression of the genes associated with oxidative stress, apoptosis, and DNA repair.	[62]
Adults	Sphere	14 nm	Citrate	0.25 and 0.8 mg/L, 20 days	-	Variations of brain and muscle AchE activity—DNA damage and alternations—variations in the expression of genes associated with oxidative stress, apoptosis, and DNA repair.	[63]
Adults	Sphere	10–20 nm and 40–50 nm	Citrate	20 µg/g/day for 28 days	-	Histological alterations in ovarian tissue	[64]
Adults	Sphere	<30 nm	-	9.7, 19.4, 29.1, 38.8, 43.65, 48.5 and 58.2 mg/L for 96 h	LC50_96 h_ = 41 mg/L	Toxicity concentration dependent—100% mortality at the highest concentration tested after 24 h—aggressive behavior after 12 h—no cytological changes—no genotoxicity.	[65]
Adults	Sphere Rod Star	16.5 nm 47 × 12 nm 42 × 16 nm	Mannose PEG	2 µL for intraperitoneal injection (5 µ/g), 120 h	-	Very low toxicity.	[66]

Abbreviations: AchE: acetylcholine esterase; CIT: citrate; CTAB: cetyltrimethylammonium bromide; dpf: days post fertilization; EM: embryo medium; hpf: hours post fertilization; MEE: 2-(2-(2-mercaptoethoxy) ethoxy); MEEE: 2-(2-(2-mercaptoethoxy)ethoxy)ethanol; MES: mercaptoethane sulfonic acid; MPA: 3-mercaptopropionic acid; PAH/SS: polystyrene-sulfate and polyallamine hydrochloride; PEG: polyethylene glycol; PVA: polyvinyl alcohol; PVP: Polyvinylpyrrolidone; PS20: Polysorbate 20; PSS polystyrene-sulfate; TMAT: trimethylammonium ethanethiol; TPPMS: triphenylphosphine monosulfonate.

**Table 2:** Effects of silver nanoparticle with different size and surface coatings in zebrafish.

Life Stage	Shape	Size	Surface Coatings	Concentration and Time of Exposure	Mortality Value vs. Doses or LC50	Results	Ref.
Embryos	Sphere	5–35 nm	PVA	10, 25, 50, 75, and 100 µg/mL, from 8-cell stage to 72 hpf	43% at 100 µg/mL	Toxicity concentration dependent—increase in mortality rate—drop in hatching rate- different phenotypic defects (absence of eyes, growth retardation, undulated notochord, larvae shorter in length)—drop in the heartbeat—decrease of the touch response.	[40]
Embryos	Sphere	3, 10, 50, and 100 nm	No coatings or TPPMS	50, 5, 0.5 and 0.05 mg/L, from 4 to 120 hpf	LC50: cAg3: 93.31µM cAg10: 125.66 µM cAg50: 126.96µM cAg100: 37.26µM	High mortality rate, time and concentration dependent—not hatched embryos—problems in the circulatory system (i.e., blood clots and hemorrhages)—high incidence of malformations, most pronounced at 120 hpf (tail malformations, pericardial edema, bent spine, and small head).	[46]
Embryos	Sphere	2–20 nm	-	1–3 µg/mL from 4 to 96 hpf	45% at 3 µg/mL	100% mortality at the highest concentration (3 µg/mL) tested—yolk sac edema and tail malformation at middle concentrations.	[61]
Embryos	Sphere	11 nm	Citrate	0.04–0.06–0.07–0.08–0.19–0.38–0.57–0.66–0.71 nM, from 8-cell stage to 120 hpf	-	Toxicity concentration dependent—increase of the mortality—different types of malformations.	[80]
Embryos	Sphere	20 or 110 nm	Citrate (C) or PVP (P)	0.8, 4, 20, 10, and 50 mg/L from 4 to 120 hpf	LC50: 20 Ag-NPs-C: 44.78 mg/mL 110 Ag-NPs-C: N/A 20 Ag-NPs-P: 42.49 mg/mL	Ag-NPs dissolved in UP and CaCl_2_ solutions more toxic than the one dissolved in EM—smaller NPs (20 nm) more toxic than larger (100 nm)—order of toxicity: 20 Ag-NPs-C > 20 Ag-NPs-P > 110 Ag-NPs-C ≈ 110 Ag-NPs-P.	[81]
Embryos	Sphere	5–20 nm	BSA	5, 10, 25, 50 and 100 mg/L from 4 to 72 hpf	LC50: 5–50 μg /mL	Toxicity concentration dependent—increased mortality rate—hatching rate delay—severe malformations (pericardial edema, twisted notochord, abnormal body axes)—bradycardia.	[82]
Embryos	Sphere	10 nm	-	3.9–8000 µg/L, from 4 to 48 hpf	No mortality	No mortality—at 24 and 48 hpf alteration in expression of genes associated with protein synthesis and oxidative phosphorylation (downregulation after 24 h of treatment and recovery after 48 h).	[83]
Embryos	Sphere	10–20 nm	DL-a-aminopropanoic acid	5, 10, 25, 50 and 100 mg/L, from 4 to 48 hpf	100% at 50–100 mg/L	Toxicity concentration dependent—100% mortality at the highest concentrations tested at 24 and 48 hpf—no alteration of heart beat rate—severe malformations (degeneration of the body, bent and twisted notochord, pericardial edema)—alteration in expression of relevant genes (*sox17, gsc, ntl, otx2*).	[84]
Embryos	Sphere	42 nm	Citrate	0.02–0.05–0.1–0.2 0.3–0.4–0.5 and 0.7 nM, from 4 to 120 hpf	-	Toxicity concentration dependent—100% of mortality at the high doses (≥ 0.2 nM)—different malformations (abnormal tail and spinal cord flexure, fin-fold deformities, eye/head defects, yolk sac edema, cardiac abnormalities) at low and middle doses (0.02–0.2 nM).	[85]
Embryos	Sphere	12 nm	Peptides: CALNNK CALNNS CALNNE	0.1, 0.2, 0.4, and 0.6 nM, from cleavage stage to 120 hpf	CALNNK^+ζ^ 33% at 0.6 nM CALNNK^−4ζ^ 77% at 0.6 nM CALNNK^−2ζ^ 42% at 0.6 nM	Toxicity concentration and charge dependent—three Ag-peptide NPs caused several malformations (cardiac malformations, severe eye abnormalities, yolk sac edema, tail, and spinal cord flexure)—positively charged NPs (Ag-CALNNK NPs^+ζ^) less toxic—more negatively charged (Ag-CALNNE NPs^−4ζ^) most toxic.	[86]
Embryos	Sphere	4 and 10 nm	Oleic acid	0.481, 0.963, 1.925, 3.850, 7.700, 11.550 and 23.100 mg/L, from 4 to 96 hpf	EC50: 4 nm: 4.120 mg/L 10 nm: 5.909 mg/L	Toxicity concentration dependent—severe malformations (bent notochord, yolk sac edema, eye, and head hypoplasia and disappearing somite)—decreased heartbeat rate, cardiotoxicity—smaller NPs (4 nm) more toxic than larger ones (10 nm)—at 24 hpf alteration in expression of neural genes (*gfap*, *neuroD*, *ngn1*, *huC*), ABCC genes and of metal responsive MTs—neurotoxicity.	[87]
Embryos	Sphere	10 nm	Polyacrylate sodium	0.03, 0.16, 0.31, 0.78, and 1.55 µg/mL of Ag, from 3 hpf to 5 dpf	LC50_96 h_: 1.18 µg/mL	Toxicity concentration dependent—increased mortality—hatching delay—reduced heartbeat rate at 48 hpf—high incidence of malformations (edema, bent tail, not depleted yolk and malformed spine)—increased ROS generation—depletion of GSH—no alteration in antioxidant enzymes activities.	[89]
Embryos	Sphere	20–30 nm	-	20 ppt, from 2.5 to 72 hpf		Alteration in the expression of genes involved in apoptosis (54%) endocytosis (10%) and immune response (18%).	[90]
Embryos	Sphere	20–40 nm	-	0.03, 0.1, 0.3, 1 and 3 ppm, from 4 to 120 hpf	5% at 3 ppm	No important changes in the mortality and hatching rates or percentage of malformations—perturbation in the behavior with hyperactivity.	[91]
Embryos	Sphere	1–20 nm	Liposomes	100–0.001 mg/L, from 12 to 72 hpf	100% at 100 mg/L	Toxicity concentration dependent—100% of mortality at the highest dose tested—scarce response to touch—decrease on the diameter of the eyes and body length—decrease of AChE and PChE activities—impaired recruitment of T-lymphocytes.	[92]
Embryos	Sphere	20 nm	Bare	0.01, 0.1, 0.5, 1 and 10 mg/L, from 6 to 21 dpf (15 days)	-	Effect on survival rate—normal body length—perturbation in genes associated with photoreception and circadian clock regulation.	[93]
Embryos	Sphere	sock-NPs: 37–165 nm spun-NPs: 15–50 nm	-	0.01–0.83 mg/L, from 6 to 72 hpf	LC50 Sock-Ag-NP: 0.14 mg/L Spun-Ag-NP: 0.26 mg/L	100% of mortality at the highest concentration tested by 24 h—reduced hatching rate and abnormal development at lower concentrations—perturbation in oxidative gene expression only for high doses (1.2 mg/L) of spun-NPs.	[94]
Embryos	Sphere	10 and 50 nm	Citrate (C) PVP (P)	0.01–100 µM, from 6 hpf to 5 dpf	-	Ag-NP-C: delay in the hatching—significant mortality—neurobehavioral changes with hyperactivity or low activity. Ag-NP-P: no hatching delay—neurobehavioral changes with hyperactivity or low activity.	[95]
Embryos	Sphere	120 nm	-	0.01, 0.1, 1 and 5 mg/L, from 2/4 to 120 hpf	-	Hatching delay and morphological defects (curved backbones) at high concentrations—upregulation of ER stress markers (*BiP* and *Synv*) and proapoptotic genes (*p53*, *p21*, *Noxa*).	[96]
Embryos	Sphere	20–30 nm	-	10 and 20 ppt, from 2.5 to 72 hpf	-	Toxicity concentration dependent—severe cardiac malformations at 72 hpf (blood pooling, pericardial edema)—abnormal notochord—apoptosis in the head—alteration of different gene expressions (activin, BMP, TGF-β, GSK3–β).	[97]
Embryos	Sphere	28 nm	Carbonate	10–200 µM, from 2 to 6 hpf	LC50: 2 hpf: 26.18 µM 4 hpf: 73.58 µM 6 hpf: 169.00 µM	Toxicity dependent on the starting time of treatments and due to the Ag^+^ action—younger embryos more sensitive.	[98]
Embryos	Sphere	46/110 nm 52/140 nm 48/155 nm 53/108 nm 78/204 nm 14/140 nm 42/77 nm	PVP TSC BIO Thiol	0.001–100 ppm, from 4 to 72 hpf	LC50_48 h_ PVP: 0.061/0.228 ppm TSC: 2.427/6.922 ppm BIO: 3.043/5.891 ppm Thiol: 0.0345/0.0845 ppm	Order of toxicity PVP > Thiol > TSC > BIO—most significant perturbations in the heart rate of embryos treated with PVP NPs—increased mortality rate and reduced hatching rate and mobility for all the tested nanoparticles.	[99]
Embryos and adults	Sphere	20–30 nm	Sodium citrate	0–10 mg/L, 48 h	LC50_48 h_ Embryos: 7.20 mg/L Adults: 7.07 mg/L	LC50_48h_ less than 10 mg/L in both embryos and adult organisms.	[100]
Embryos/Adults	Sphere	5 nm	PVP/PEI	Embryos: 10 μg/L–10 mg/L for 5 days Adults: 100 ng/L for 3 weeks	LC50_120 h_: 50 μg/L	Embryos: 100% of mortality at high doses (0.1 mg/L) after 24 h of exposure—significant effect on the hatching rate. Adult: vacuolization of the liver- reduction of fecundity—resulting embryos with malformations.	[101]
Embryos	Sphere	25–75 nm	PVP	0–100 mg/L, from 24 to 120 hpf	LC50_24–48 hpf_: 51 mg/L	Toxicity increase during the development—less sensitivity compared to AgNO_3—_alteration in expression of metal ion chaperone metallothionein.	[102]
Embryos	Sphere	20–40 nm	-	10 and 500 µg/mL, from 3/3.5 to 72 hpf	Ec-Ag-NP: 154 µg/mL SA-Ag-NP: 142 µg/mL ST-Ag-NP: 125 µg/mL BT-Ag-NP: 185 µg/mL	Toxicity dependent on concentration biogenicity and exposure time—hatching delay—decreased heartbeat rate—malformations (yolk, notochord, gastrointestinal lumen)—apoptosis in the tail area and head—ROS production.	[103]
Embryos	Sphere	4 and 10 nm	Oleic acid	0.481, 0.963 and 1.925 mg/L, from 4 to 96 hpf	-	4 nm: delayed yolk sac absorption, reduced body length—alteration in expression of *Pxmp2* and *HIF4*. 10 nm: no significant adverse effects.	[104]
Embryos	Sphere	9 and 30 nm	PVP and EG	10 and 20 µg/mL from 4 to 120 hpf	9 nm: 100% at 20 µg/mL 30 nm: 60% at 20 µg/mL	Toxicity size and concentration dependent—increased mortality rate—decreased hatching rate and heartbeat rates—high incidence of malformations (tail malformation, yolk sac edema, axial deformity, pericardial edema, eye defects).	[105]
Embryos	Sphere	12 and 49 nm	PVP GA	2 mg/L Ag	PVP-Ag-NPs in WP: 8% PVP-AG-NPs in WPS: 10% PVP-Ag-NPs in W: 74% GA-Ag-NPs in WP:19% GA-Ag-NPs in WPS: 6% GA-Ag-NPs in WS: 35% GA-Ag-NPs in W: 81%	Mortality rates of zebrafish treated with of GA-Ag-NPs or PVP-Ag-NPs in three different matrices lower with respect to GA-Ag-NPs or PVP-Ag-NPs in water-sediments protective against the GA and PVP-Ag-NPs toxicity.	[106]
Embryos	Sphere	10 and 35 nm	Bare Citrate	5, 50, 500, 5000, and 25.000 µg/L, from 1.5 to 48 hpf	10 nm Ag-NPs: 79% at 25.000 µg/L CIT-Ag-NPs: 14% at 25.000 µg/L	Toxicity size and concentration dependent—35 nm Ag-NPs more toxic than 10 nm—coated NPs less toxic than the uncoated ones. 35 nm: highest mortality during gastrulation—yolk sac damage—high cell death in the yolk sac.	[107]
Embryos	Sphere	15, 35, and 80 nm	Bare DIS PVP	0.05–1000 µM, from 4 to 96 hpf	EC50: Ag-NPs: 1.95 µM DIS-Ag-NPs. 0.83 µM PVP-Ag-NPS: 1.50 µM	Decreasing toxicity order: DIS-nAg > PVP-nAg > Bare-nAg.	[108]
Embryos	Sphere	40 nm	Citrate	0.4 mg/L, from 4 to 72 hpf	-	Hypopigmentation—reduced xanthocytes and melanocytes—alteration in expression of xanthophone (*gch2*) and melanophore genes (*mitfa* and *dct*) and genes associated with the development of neural crest (*foxd3* and *pax7*)	[109]
Embryos	Sphere	10 and 40 nm	Citrate	0.2, 0.4, 0.75 and 1 mg/L, from 4 to 96 hpf	-	40 nm: Mortality and incidence of malformations (cardiac defects and yolk sac edema) concentration dependent—high incidence of malformations—downregulation of hemoglobin complex genes (*hbae1*, *hbbe1*, *hbbe2*, *scf4* and *alas2*)—inhibition of erythroid differentiation—no effects on blood vessel formation or mesodermal specification10 nm: low incidence of malformations.	[111]
Adults	Sphere	< 30 nm		15.5, 18.6, 21.7, 24.8, 27.9 and 31 μg/L for 96 h	LC50_96 h_: 24.5 μg/L	Toxicity concentration dependent—100% mortality at the highest concentration tested after 12 h—aggressive behavior after 6 h—cytological changes in gills and liver (pyknotic nuclei, cell membrane damage)—high levels of AST and ALT—production of ROS—alteration in expression of genes associated with immune response (C/EBP, IL1β and LYZ, MPO, TLR22, NF-κB, and TLR4) and oxidative stress (MTF1, HSP70).	[65]
Adults	Sphere	5–20 nm	-	30, 60 and 120 mg/mL, 24 h	-	Cellular disruption in the liver tissue (pyknosis and chromatin condensation)—increase of MDA and GHS levels—alteration in the expression of oxyradical scavenging enzymes (Cat and GPx1 a)—DNA damage—upregulation of proapoptotic genes (*Bax* and *Noxa*).	[110]
Adults	Sphere	81 nm	PVP	18, 36, 54, 72, 89, 107, 125, and 143 μg/L, 48 h	LC50_48 h_: 84 μg/L	Toxicity concentration dependent—100% mortality after 24 h exposure to the highest concentration tested (143 μg/L)—extravasations of blood in the anterior ventral surface of the body—lost equilibrium—increased respiratory rate.	[112]
Adults	Sphere and polygon	11–45 nm17–40 nm	-	for 28 days	LD50_96 h_: chemically synthesized Ag-NPs: 80 µg/L biologically synthesized Ag-NPs: 400 µg/L	Biologically synthetized Ag-NPs less toxic in comparison to both the chemical counterparts and Ag NO_3_—histological damage in ovarian tissue (mild atresia).	[113]
Adults	Sphere	22–26 nm	PVP	0.1 ppm, 15 days	-	Sulfidation mitigated the toxicity—mitigation of induced oxidative stress—protection of liver and brain biochemical enzymes.	[115]
Adults	Sphere	20–30 nm	-	1 mg/L, 24 h and 48 h	-	No alteration in gill filament—dramatic alterations in global gene expression patterns, particularly genes associated with mitogenesis, proliferation, and apoptosis.	[116]
Adults	Sphere	3 nm	Citrate	5, 15, 25, or 50 µg/L, 28 days	No mortality	No mortality—no morphological alterations in gills—significant alterations in the expression of genes associated with morphogenesis and developmental processes.	[117]
Adults	Sphere	25 nm	-	8, 45, and 70μg/L for 30 days	-	Damage in gills structure (lamellar fusion, subepithelial edema)—necrosis of intestinal villi—high expression of MTs1.	[118]
Adults	Sphere	25–100 nm	Bare	5, 10, 20, 25, and 30 mg/L for 4 days—2 and 4 mg/L for 1–3 weeks	LC50_96 h_: 16.76 mg/L	Mortality concentration and time dependent—decrease of gill Na^+^/K^+^ ATPase activity only after 14 and 21 days—decreased erythrocyte AChE activity after 4 d—alterations of electrolyte levels in a short time—increase of plasma cortisol and glucose levels.	[119]
Adults	Sphere	5–50 nm	-	23.7, 47.4, 142.2, 237, 284.4, 331.8 µg/L, for 96 h 71.1 µg/L for 14 days	LC50_96 h_: 142.2 µg/L	Exposure for 96 h: 100% mortality at the highest concentration tested—increase in respiratory rate (respiratory toxicity). Exposure for 14 days: oxidative stress with morphological changes in liver and gills—genotoxicity with misregulation of genes associated with immune and stress responses.	[120]
Adults	Sphere	50.7 ± 1.8 nm	-	10, 33 or 100 μg/L for 35 days	-	Alteration of gut microbiota composition in terms of richness and diversity only in male fish—Male: abundance of *Proteobacteria*—low *Fusobacteria*, *Cetobacterium* and *Aeromonas*.	[121]
Adults	Sphere	20–70 nm	-	10, 33, and 100 mg/L for 5 weeks	No mortality	No mortality—both in testis and ovary ROS generation—decrease of fecundity—increase of apoptotic cells in gonads—alteration in the expression of genes linked to mitochondrion-mediated apoptosis (*bax*, *bcl-2*, *caspase-3,* and *caspase 9*).	[122]
Adults	Sphere	10	PVP	1 or 5 mg/kg by injection for 24 h	No mortality	No mortality—damage in kidneys, spleen, heart, and gall bladder—alteration in the expression of IL-1 and TNFα, caspase 6 and 9.	[123]
Adults	Sphere	20–110 nm	Citrate	1 ppm, for 4 h, 4 days, or 4 days plus 7 days	-	Size-dependent toxicity—morphological changes in intestines and gills—decrease of Na^+^/K^+^ ATPase activity. 20 nm: hyperplasia, injury, fusion in the gill tissue—not significant effect in the intestine. 110 nm: less significant changes in the gills—vacuolization and damage in the intestine.	[124]

Abbreviations: AchE: acetylcholine esterase; AST: aspartate transaminase; ALT: aka alanine aminotransferase; BIO: biocompatible gelatin; BSA: bovine serum albumin; Cat: catalase; C/EBP: CCAAT-enhancer binding protein; DIS: dispersant; dpf: days post fertilization; EG: Ethylene glycol; EM: embryo medium; ER: endoplasmatic reticulum; GA: gum arabic; GPx1 a: glutathione peroxidase 1 a; GSH: glutathione; HIF4: hypoxia inducible factor 4; HSP70: heat-shock protein 70; hpf: hours post fertilization; IL1: interleukin 1; IL1β: interleukin 1 beta; LC50: lethal concentration, 50%; LYZ: lysozyme; MDA: Malondialdehyde; MTs: metallothioneins; MT1 s: metallothioneins 1; MTF1: metal transcription factor 1; NF-κB: nuclear factor-κB; PChE: pseudocholinesterase; PEI: polyethylenimine; PVA: polyvinyl alcohol; PVP: polyvinylpyrrolidone; Pxmp2: perosomial membrane protein 2; ROS: reactive oxygen species; Thiol: 16-Mercaptohexacanoic Acid; TPPMS: triphenylphosphine monosulfonate; TLR22: Tolllike receptor 22; TNFα: Tumor necrosis factor 1; TSC: Trisodium citrate; UP: ultrapure water; W: water only; WP: water + plants; WPS: water + plants + sediment; WS: water +sediment.

## Data Availability

Not applicable.
